# Fulminant eosinophilic myocarditis and doxycycline administration: a case report

**DOI:** 10.1093/ehjcr/ytae587

**Published:** 2024-11-15

**Authors:** Alexandre Salaûn, Georges Tarris, Bernard Bonnotte, Yves Cottin

**Affiliations:** Department of Cardiology, Dijon Bourgogne University Hospital, France; Department of Anatomical Pathology, Dijon Bourgogne University Hospital, France; Department of Internal Medicine, Dijon Bourgogne University Hospital, 14 rue Gaffarel, 21079 Dijon Cedex, France; Department of Cardiology, Dijon Bourgogne University Hospital, France

**Keywords:** Eosinophilic myocarditis, Doxycycline, Parasitic lysis, Ventricular electrical storm, Cardiogenic shock, Case report

## Abstract

**Background:**

Eosinophilic myocarditis is a life-threatening condition with a heterogeneous clinical presentation and aetiology. Cases of drug-induced or parasitic myocarditis have been reported but there is scant literature on the involvement of treatments, such as doxycycline, and eosinophil degranulation due to parasitic lysis.

**Case summary:**

Here, we report the case of a 59-year-old man without a relevant past medical history who developed a skin rash with hepatic cytolysis and mild eosinophilia. No aetiology was found despite an exhaustive work-up, but a parasitic infestation was suspected in view of the patient’s daily contact with freshwater environments. A few days after doxycycline administration, the patient’s clinical state worsened rapidly leading to a ventricular electrical storm-related cardiogenic shock requiring mechanical support. After initiation of high-dose corticosteroid therapy, cardiac function normalized promptly allowing for withdrawal of the mechanical support. An endomyocardial biopsy led to the diagnosis of eosinophilic myocarditis, which was congruent with the cardiac magnetic resonance imaging data.

**Discussion:**

The main aetiologies reported for eosinophilic myocarditis are often allergic reactions, such as DRESS syndrome in developed countries, or infections, especially due to parasites in other countries. Drugs such as tuberculosis medications, antipsychotics, and antiepileptics have been implicated, as well as antibiotics, e.g. minocycline, but there has been no case of doxycycline-related eosinophilic myocarditis reported to date. Parasitic lysis is known to induce the activation of eosinophils and their on-site degranulation but no case has been reported on myocarditis due to parasitic lysis after administration of antiparasitic drugs.

**Learning points**

- Eosinophilic myocarditis is a life-threatening condition requiring urgent high-dose corticoid therapy.
- Drug-related cases have been described but none following doxycycline administration.
- To date, parasitic lysis has not been reported as an aetiology of eosinophilic myocarditis.

## Introduction

The cardiac effects of hypereosinophilia vary widely, from asymptomatic patients to fulminant necrotizing eosinophilic myocarditis. Currently, the diagnosis is based on imaging techniques in addition to endomyocardial biopsy (EMB). Eosinophil-related disorders can be treated aetiologically, but also with initial high-dose corticosteroid therapy to limit lesion extension and the subsequent risk of developing restrictive heart disease. Here, we present a case of eosinophilic myocarditis confirmed by EMB, most likely related to an allergic reaction following administration of doxycycline.

## Summary figure

**Figure:**
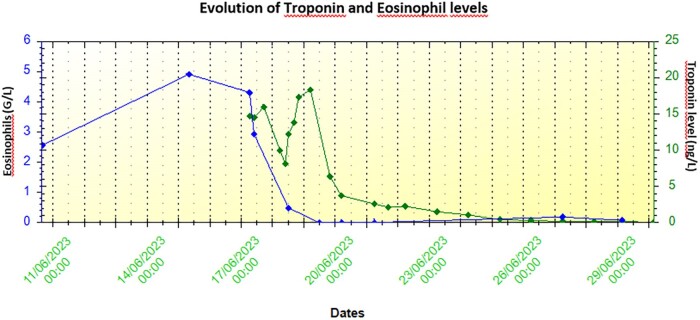


### Case report

**Figure 1:**
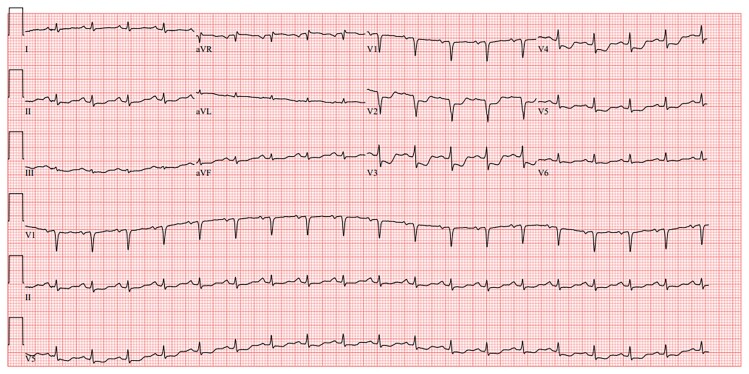
Electrocardiogram showing sinus tachycardia, with QS complexes in V_1_–V_2_, and subendocardial injury current from V_2_ to V_5_.

**Figure 2:**
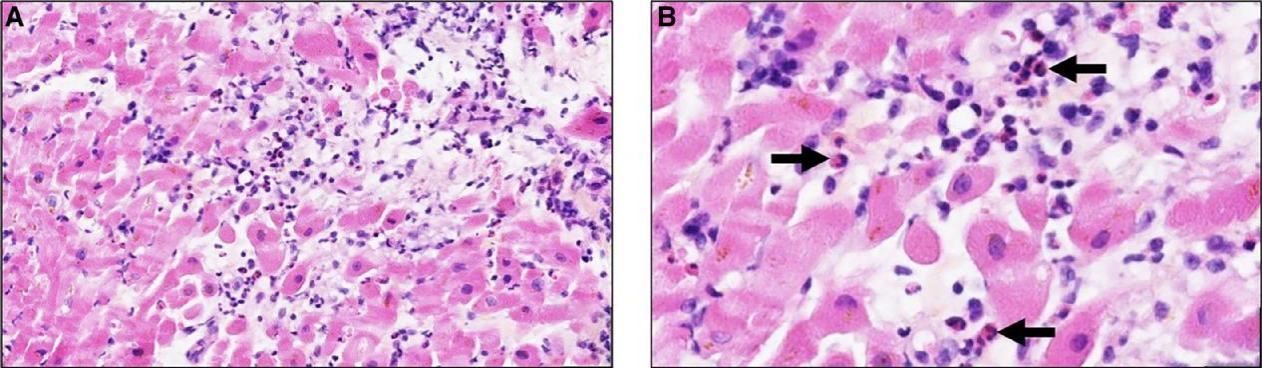
Histopathology of an endomyocardial biopsy in a patient suffering from cardiogenic shock. (*A*) (HES, ×100): partial destruction of myocardial fibres is observed, associated with a heavy inflammatory infiltrate with numerous eosinophils, scarce macrophages and lymphocytes. (*B*) (HES, ×300): at higher magnification, the numerous eosinophils are observed in the inflammatory infiltrate (arrows).

**Figure 3:**
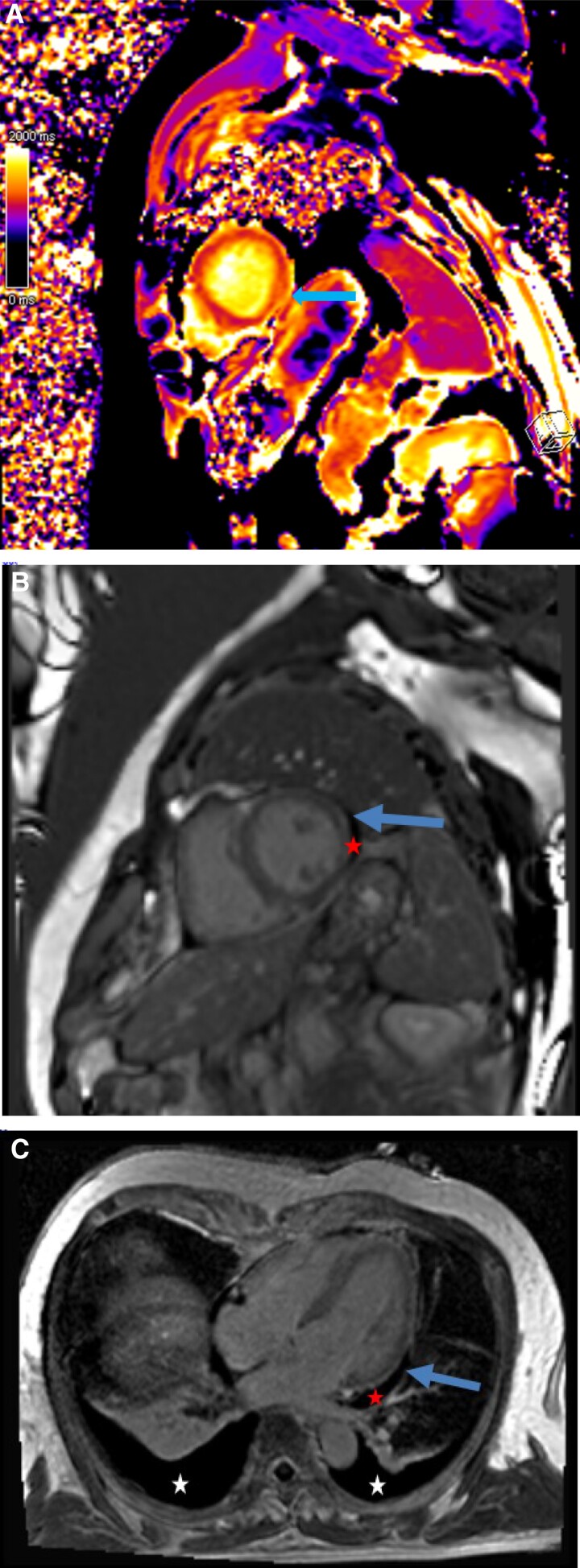
Cardiac MRI displaying subepicardial gadolinium chelate enhancement particularly in the anterolateral and basal inferolateral segment (blue arrow) with pericardial effusion (red star) in late phase-sensitive inversion recovery (PSIR) acquisition in short-axis (*B*) and apical four-chamber long-axis (*C*) views. Native T1 mapping sequence displays an increase in T1 relaxation time up to 1366 ms in the subepicardial area in the same segment (*A*, blue arrow), whereas T1 relaxation time is evaluated at 1200 ms in safe myocardium (T1 value of normal myocardium is 950–1000 ms). Acute myocyte lesions are corroborated by mapping, but the extension of fibrosis cannot be precisely evaluated considering the oedematous component. Note the bilateral pleural effusion (white star) in the apical four-chamber long-axis (*C*) view in this patient with lightly altered cardiac function (left ventricular ejection fraction = 40%). No atrial dilation or intraventricular thrombus is found.

The case was a 59-year-old male patient with no relevant past medical history or cardiovascular risk factors other than age, sex, and occasional alcohol consumption. The patient was a supplier of fishing equipment and had regular contact with freshwater environments. Over the previous 2 months, he had been experiencing an altered general condition including a generalized maculopapular rash and transaminase elevation for which no infectious or autoimmune aetiology had been identified despite an exhaustive work-up. The clinical exam showed nothing abnormal at first. The medical team suspected an infection due to his regular contact with freshwater environments; however, the subsequent introduction of doxycycline resulted in marked hypereosinophilia associated with rapid haemodynamic deterioration alongside dyspnoea, hypotension, and sinus tachycardia with non-reciprocal precordial ST-segment depression (*Figure 1*). Troponin levels rose to 15 000 ng/L (normal < 53.00 ng/L) and a transthoracic echocardiogram performed at that time revealed an impaired left ventricular ejection fraction (LVEF) of 40% with diffuse left ventricular hypertrophy reaching 14 mm at the septal level with an oedematous appearance of the myocardium, as well as severe hypokinesia of the lateral wall. The patient was transferred to the cardiology unit and underwent coronary angiography, which revealed no coronary anomaly that would explain the clinical presentation. The ventricular arrhythmias were refractory to the first line drugs, i.e. intravenous beta-blockers (landiolol) and amiodarone, i.v. lidocaine, even after deep sedation and double stellate ganglion block. The patient then developed cardiogenic shock, followed a few hours later by cardiorespiratory arrest requiring veno-arterial ECMO (extracorporeal membrane oxygenation) and intra-aortic balloon counterpulsation with simultaneous administration of amiodarone, Xylocaine, and a stellate ganglion block. Given the increase in eosinophil count to 4.9 g/L (normal < 0.5 g/L) as well as the elevation in troponin levels, in the hypothesis of myocarditis, the patient received high-dose intravenous corticosteroid therapy with methylprednisolone sodium succinate 1 g for 3 days. The patient’s haemodynamic status improved, allowing mechanical support to be withdrawn after 3 days. Cardiac MRI was then performed, showing 40% LVEF with subepicardial contrast enhancement, which was suggestive of an episode of basal inferolateral myocarditis (one segment) (*Figure 2*). Unfortunately, the quality of T2-TIRM (turbo inversion recovery magnitude) was too poor to be included in the case but the diagnosis of myocarditis was supported by cardiovascular magnetic resonance according to the Lake Louise criteria. An EMB was performed, revealing significant eosinophilic infiltrate thus confirming the diagnosis of eosinophilic myocarditis (*Figure 3*).

After discharge from intensive care, the patient’s haemodynamic parameters returned to the normal range. Left ventricular ejection fraction was 50%, with disappearance of kinetic abnormalities. There was no recurrence of ventricular rhythm disorder. However, given the high risk of subsequent rhythm disturbances in this patient, an implantable cardioverter-defibrillator (ICD) was placed in addition with medical management using beta-blockers and angiotensin-converting enzyme inhibitors. After 6 months’ follow-up, the patient showed no relapse of the disease, no myocarditis, and no hypereosinophilia and received only beta-blockers.

## Discussion

The incidence of myocarditis is poorly established due to diagnostic difficulties (heterogeneous clinical presentation, lack of access to myocardial MRI and/or EMB), but autopsy studies in young sudden cardiac death patients have shown that 2%–42% of these patients had myocarditis.^1–4^ Among the aetiologies of myocarditis, hypereosinophilia is a rare and serious condition, with an in-hospital mortality rate of 10% and of 70% at 3 years.^1^ Although, cardiac MRI is sufficient to establish the diagnosis of acute myocarditis when the Lake Louise criteria are met, EMB remains the gold standard for establishing a definitive aetiological diagnosis.^5,6^ In the specific case of eosinophilic myocarditis, EMB has immediate and specific therapeutic consequences if the diagnosis is established, but it should also be emphasized that histological abnormalities disappear very rapidly with treatment.^5^ However, EMB is not very sensitive (∼50%) since infiltration is generally focal.^7^ Furthermore, if the patient presents with pericardial effusion, cytology of the pericardial effusion may show a large number of eosinophils associated with elevated concentrations of interleukin (IL)-5 and IL-13 in the pericardial effusion and blood.^7^ Consequently, the joint recommendations of the American and European cardiology societies propose reserving this procedure for the most severe forms responding to immunosuppressive treatment, including acute necrotizing eosinophilic, giant cell, and immune checkpoint inhibitor myocarditis.^8^ In our case, EMB coupled with cardiac MRI confirmed the diagnosis and enabled treatment to be started quickly. Eosinophilic myocarditis has a wide range of aetiologies, including viral infections, parasitic infestations, allergic reactions, vasculitis, mainly eosinophilic granulomatosis with polyangiitis (EGPA), lymphoid or myeloid haemopathies, solid neoplasia, and lymphoid or myeloid essential hypereosinophilic syndrome (HES).^9,10^ Among the 179 histologically proven cases of eosinophilic myocarditis, hypersensitivity was the most frequently reported condition (34%), followed by EGPA (13%), HES (8%), and *Toxocara canis* infection (5%).^11^ The aetiology or associated condition most frequently encountered worldwide is parasitic infection, while allergic reactions are the most common cause in developed countries. Identifying the aetiology or condition associated with eosinophilic myocarditis is important for specific treatments, as the use of albendazole when associated with *T. canis* infection, cyclophosphamide in EGPA or imatinib in the PDGFRA-associated myeloproliferative variant of HES.^12^ However, 35% of cases had an idiopathic eosinophilic myocarditis although a thorough aetiological work-up was performed.^11^ In our case, autoimmune and ANCA-associated vasculitis, viral, and neoplastic aetiologies were ruled out by clinical, autoantibody, and negative serological investigations and normal imaging, the last two likely hypotheses being: parasitic infestation with parasite lysis after doxycycline administration or hypereosinophilia associated with drug toxicity or allergy after doxycycline administration. Numerous cases of hypereosinophilia associated with acute or chronic myocarditis during parasitic infections have been published. Parasitic lysis induced by immune cells can promote the activation and degranulation of eosinophilic polynuclei, resulting in the release of their cytotoxic mediators, including cation-binding protein, which is highly toxic to the myocardium. However, no parasitic infection could be diagnosed despite extensive research in our patient including EBM. In the literature, several cases of eosinophilic myocarditis have been reported following drug administration. Although no myocardial damage associated with doxycycline has been reported to date, the diagnosis of DRESS syndrome seems the most likely. Treatment of acute eosinophilic myocarditis includes management of acute heart failure and following mechanical or rhythm complications associated with eosinophil elimination. Approximately 30 cases have been published in the literature in which high-dose systemic corticosteroid therapy was instituted as soon as eosinophilic myocarditis was suspected, and most patients made a full clinical recovery.^11–14^ No randomized controlled trials are available to date, thus the current dosing regimen remains empirical. In cases of corticosteroid resistance or dependence, immunosuppressants or anti-IL-5 monoclonal antibodies are introduced with favourable outcomes.^11,14,15^ In our case, the worsening of hypereosinophilia after introduction of doxycycline argues in favour of an immuno-allergic reaction to this drug. Regarding follow-up, cardiac MRI is now the preferred approach to monitoring these patients. In our case, a follow-up MRI was not feasible at an early stage due to the recent implantation of the ICD, and therefore 18-FDG positron emission tomography was performed 1 month after the diagnostic MRI, showing no cardiac fixation.

## Conclusion

Eosinophilic myocarditis remains a rare condition that is potentially under-diagnosed in insidious forms, and which has a poor prognosis depending on the degranulation activity of eosinophils *in situ*. This degranulation may be exacerbated by the aetiology and corresponding treatment, as in the case of parasitic lysis induced by an anti-infective agent, provoking a severe eosinophilic reaction. Whatever the aetiology of hypereosinophilia, once the cause has been identified, aetiological treatment must be combined with therapy that rapidly eliminates eosinophils, such as corticosteroids, to which anti-IL-5 and anti-IL5R can be added. Patient follow-up is necessary because of the risk of relapse, particularly when no cause has been identified. However, further studies are needed to better understand the pathophysiology of hypereosinophilic myocarditis and to improve therapeutic options.

## Data Availability

The data underlying this article will be shared on reasonable request to the corresponding author.

## Lead author biography

Alexandre Salaûn is a cardiology resident currently working in the Cardiology Intensive Care Unit in the François-Mitterand Hospital, Dijon in France.

## Notes

**Consent:** The authors confirm that written consent for submission and publication of this case report including images and associated text has been obtained from the patient according to the COPE guidelines.

**Funding**: This research did not receive any specific grant from funding agencies in the public, commercial, or not-for-profit sectors.
